# Optical Labeling with Artificial Intelligence Using Infrared-Responsive Functional Textiles

**DOI:** 10.1021/acsami.5c13595

**Published:** 2025-10-11

**Authors:** Yi-Ting Tsai, Chi-Wei Wu, Ren-Jei Chung, Gabriel Nicolo A. De Guzman, Guan-Jie Li, Julius L. Leaño, Mu-Huai Fang

**Affiliations:** † Research Center for Applied Sciences, 38017 Academia Sinica, Taipei 11529, Taiwan; ‡ Department of Chemical Engineering and Biotechnology, 34877 National Taipei University of Technology, Taipei 10608, Taiwan; § Department of Science and Technology, 610100 Philippine Textile Research Institute, Taguig City 1631, Philippines; ∥ Isuzu Optics Cooperation, Hsinchu 30288, Taiwan

**Keywords:** smart textiles, short-wave infrared, hyperspectral imaging, AI recognition, convolutional neural network

## Abstract

Functional textiles integrated with image recognition systems hold significant potential for advanced applications. However, conventional visible light dyes and colorants can be detected under normal lighting conditions and are difficult to conceal within materials. In this study, we introduce a sustainable and intelligent textile platform by integrating SiO_2_/Al_2_O_3_-coated hydrophilic Ag_2_S quantum dots into natural pineapple leaf fiber (PALF). This biogenic composite exhibits excellent photostability and water compatibility, with concealed optical functionality detectable only under short-wave infrared imaging. Unlike conventional visible dyes, the embedded emission remains invisible under ambient conditions, enabling covert information encoding. To achieve material-level recognition, a convolutional neural network (CNN) was trained on hyperspectral imaging data and successfully identified optically functionalized fibers with 82% accuracy. This was demonstrated even in millimeter-scale yarn samples (∼0.6 cm). This work exemplifies the synergy between renewable materials, nanophotonics, and artificial intelligence, offering practical utility in secure labeling, anticounterfeiting, and interactive textiles.

## Graphical abstract

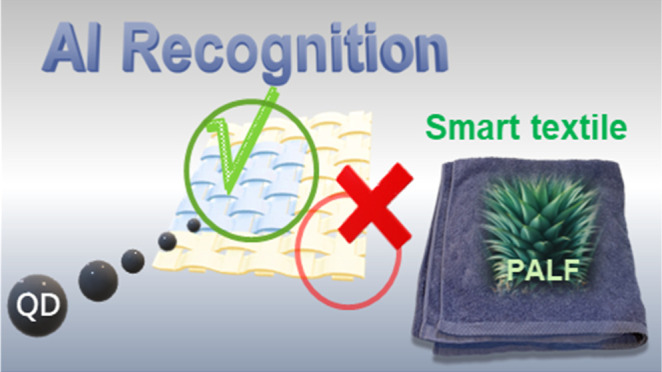

## Introduction

Smart textiles are evolving from conventional wearables into platforms with integrated sensing, communication, and identification capabilities.^1–4^ While electronic-based smart textiles offer advanced functionalities, they raise environmental and health concerns due to their complexity and electronic waste.^5,6^ Alternatively, embedding functional markers directly into fibers provides a sustainable route for information storage, anticounterfeiting, and optical sensing.^7–10^

In 2023, Patti et al. reviewed the incorporation of fluorescence into smart textiles, highlighting the potential applications of optical interaction technologies.^11,12^ Recent advances demonstrate that natural textile fibers (NTFs) derived from agricultural waste, such as pineapple leaf fiber (PALF) and lyocell, serve as eco-friendly substrates for functional textiles.^13–15^ Lyocell, a regenerated cellulose, offers high moisture absorption and scalability. Both fibers provide strong breathability and dye uptake along with suitable mechanical strength, making them ideal for integration into smart textile systems.^16–19^ From a sustainability perspective, there is a growing interest in carbon quantum dots (CDs) and silver sulfide (Ag_2_S) given their nontoxic and environmentally benign properties.^20–25^ Additionally, Ag_2_S exhibits tunable short-wave infrared (SWIR) emission within the invisible light spectrum in the 1100–1700 nm range, making it ideal for creating covert markings in smart textiles.^26–29^

To address these issues, we adopted a sol–gel method to prepare a SiO_2_/Al_2_O_3_ monolith (SAM) coating using a single precursor, disec-butoxyaluminoxytriethoxysilane (DBATES).^30^ This compact layer shields Ag_2_S quantum dots (QDs) from moisture and oxygen to enhance their environmental stability. By replacing the initial hydrophobic ligand with a hydrophilic interface, we obtained Ag_2_S@SAM with improved adhesion to the PALF-lyocell yarn. To ensure uniform particle size, we applied a ball-milling process to Ag_2_S@SAM and generated a composite defined as Ag_2_S@SAM-B. This nanocomposite was chemically grafted to fibers using carboxymethyl cellulose (CMC) as the dispersant and citric acid (CA) as the cross-linking agent.^31^ The treated yarns were processed by standard spinning techniques to yield stable and fluorescent textile threads. Unlike physical printing, this method enables the robust integration of Ag_2_S QDs within the fiber structure. We further demonstrate the use of hyperspectral infrared imaging for detecting SWIR markers and potential implementation in AI-based classification systems, as shown in Figure 1.

**Figure 1:**
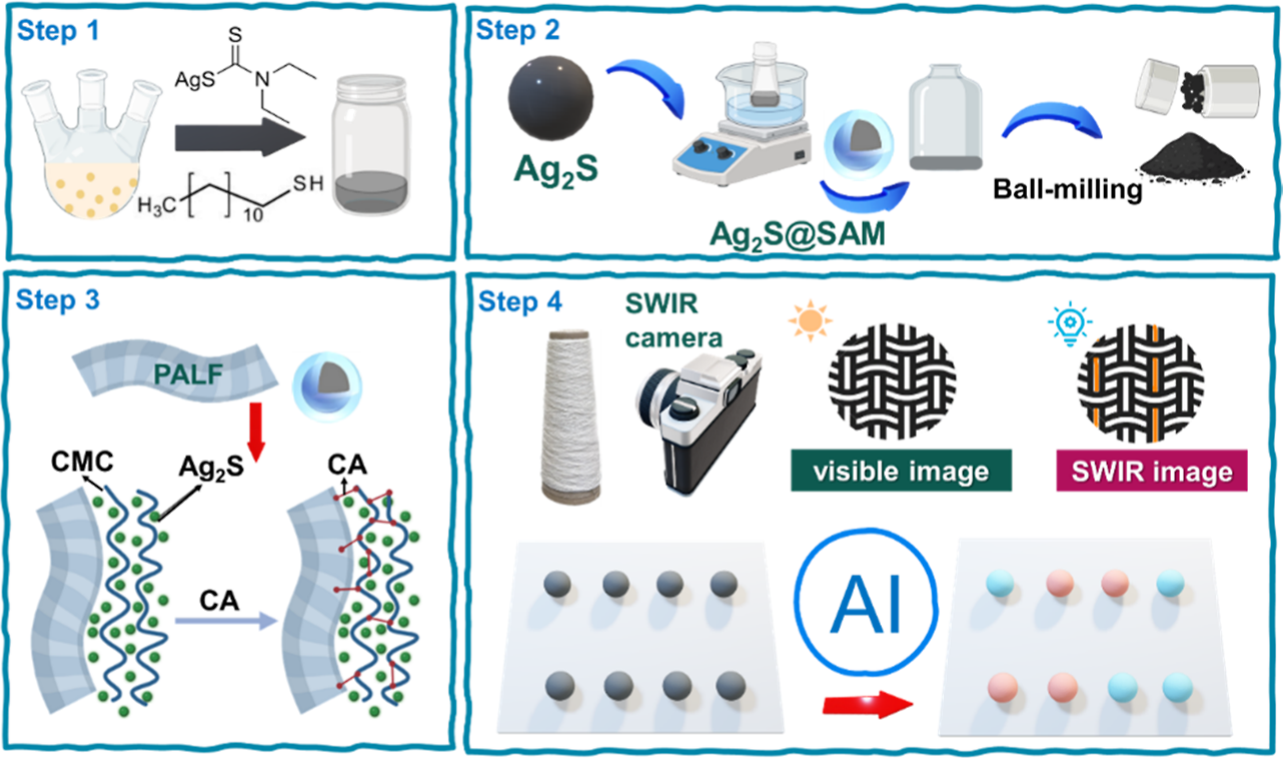
Preparation and assembly of the multifunctional SWIR quantum-dot-embedded smart ananas comosus leaf fiber-containing textile.

## Results and Discussion

### Ag_2_S Structure and Optical Analyses

The primary objective of this study is to develop hydrophilic Ag_2_S materials and integrate them with PALF-lyocell textile fibers to create advanced smart textiles with enhanced functional properties. To ensure that the Ag_2_S crystal structure remained intact throughout the synthesis process, synchrotron high-resolution X-ray diffraction (HRXRD) was employed. The diffraction patterns obtained were compared to the standard crystallographic data COD-9000253 for monoclinic Ag_2_S. The Ag_2_S crystallizes in a monoclinic structure in the *P*2_1_/*c* space group with two distinct Ag sites, as illustrated in Figure S1. The HRXRD spectra of Ag_2_S, Ag_2_S@SAM composites, and Ag_2_S@SAM-B confirmed that the crystal structure of Ag_2_S remained consistent with the standard monoclinic pattern throughout the processing steps. The broad diffraction peak observed between 7.5° and 17.5° indicates the presence of an amorphous phase attributed to the SAM coating. The diffraction data (Figure 2a) showed no evidence of a phase transition or structural degradation, confirming the robustness of the synthesis process.

**Figure 2:**
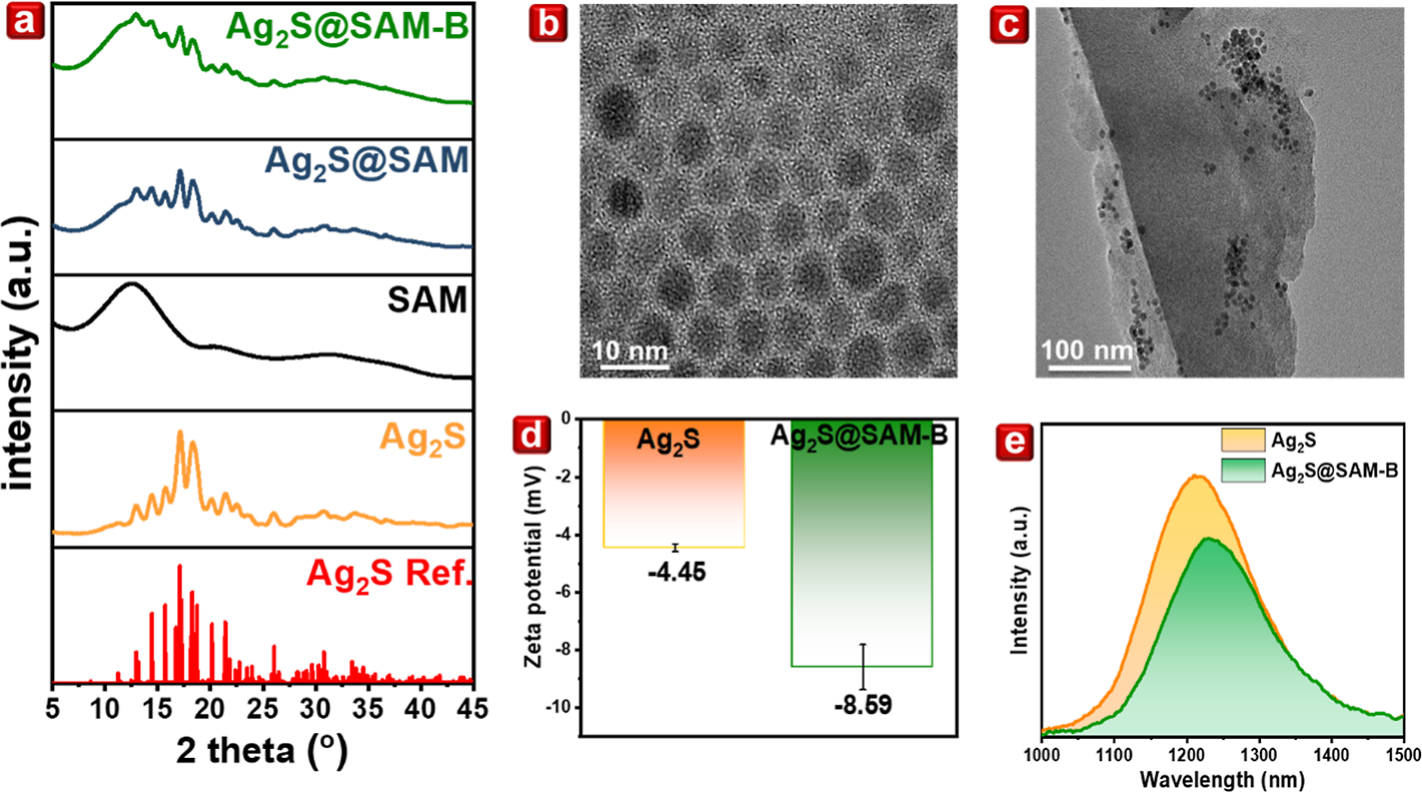
(a) High-resolution synchrotron XRD patterns of the Ag_2_S, SAM, Ag_2_S@SAM, and Ag_2_S@SAM-B. High-resolution TEM images of (b) Ag_2_S and (c) Ag_2_S@SAM. (d) Zeta potential analysis of Ag_2_S and Ag_2_S@SAM-B. (e) PL spectra of Ag_2_S and Ag_2_S@SAM-B under 450 nm excitation.

The high-resolution transmission electron microscopy (HRTEM) images of Ag_2_S and Ag_2_S@SAM, shown in Figure 2b,c, reveal the distribution of particles. The Ag_2_S particle size distribution indicates an average particle size of 6.7 ± 0.5 nm. After the SAM coating process, the particle size increased slightly to 8.3 ± 1.0 nm (Figure S2). This size increase is attributed to the continued growth of the Ag_2_S crystal during the SAM synthesis, which occurs before the complete formation of the SiO_2_/Al_2_O_3_ protective layer. QDs within the coating layer display a random distribution, maintaining their overall morphology. To verify the elemental composition and distribution within the Ag_2_S@SAM composite, energy-dispersive X-ray spectroscopy (EDS) mapping was performed on the TEM images. As shown in Figure S3, the elements silver, sulfur, aluminum, silicon, and oxygen are represented by light blue, yellow, green, dark blue, and red, respectively. The distribution of aluminum, silicon, and oxygen surrounding the silver and sulfur elements confirms that the SAM protective layer successfully encapsulated the Ag_2_S QDs. Additionally, HRTEM images at a higher magnification (Figure S4a) reveal lattice fringes of the Ag_2_S crystal, indicating that Ag_2_S retained its crystallinity. Fast Fourier transform (FFT) analysis of the red-framed region in the HRTEM image (Figure S4b) shows diffraction patterns with *d*-spacings. While HRTEM provided detailed nanoscale structural insights, the SAM coating process produced random microsized amorphous phase materials. To assess the material at a macroscopic level, we used scanning electron microscopy (SEM). Before ball milling, the Ag_2_S@SAM composite exhibited an irregular morphology with particle breakage and stacking, resulting in a broad particle size distribution of 46.3 ± 52.9 μm (Figure S5a,b). After ball milling, the particle size was significantly reduced to 3.5 ± 5.7 μm, with a much narrower particle size distribution, as shown in Figure S5c,d. This reduction in particle size also improved the material’s dispersibility in solvents, confirming the importance of ball milling in achieving particle size uniformity and enhanced stability in the composite material.

To effectively use Ag_2_S@SAM-B in fluorescent smart textiles, assessing its optical properties and hydrophilicity after surface modification is essential. The presence of surface functional groups was confirmed by FTIR spectroscopy, with detailed spectra presented in Figure S6.^32^ These results confirm that dodecanethiol’s original long carbon chain ligands on the surface of Ag_2_S quantum dots have been successfully converted to a hydrophilic SAM surface. This enhanced hydrophilicity is expected to improve the compatibility of the composite material with natural textile fibers, thereby facilitating its integration into smart textile systems. Furthermore, zeta potential measurements were used to assess the surface charge of Ag_2_S and Ag_2_S@SAM-B before and after surface modification. The zeta potential of Ag_2_S quantum dots increased from −4.45 to −8.59 mV after SAM modification, as shown in Figure 2d, indicating an increase in the negative surface charge. This variation is attributed to negatively charged hydroxyl (−OH) groups forming during the hydrolysis and condensation reactions of the SAM precursor, further confirming the successful modification of SAM.^33^ Moreover, the zeta potential is also a useful indicator of the particle dispersion in solution. A higher absolute zeta potential value suggests reduced particle agglomeration and improved dispersion stability. The results from FTIR spectroscopy and zeta potential analysis provide compelling evidence that the surface modification of Ag_2_S QDs enhances their hydrophilic properties and improves their dispersibility, making them more suitable for application in smart textile technologies.

The unique broadband luminescence properties of Ag_2_S in the SWIR range provide an effective strategy for developing fluorescent smart textiles for security and covert applications. The absorption spectrum (Figure S7) demonstrates a broad absorption range covering visible to infrared wavelengths. The photoluminescence (PL) spectrum in Figure 2e illustrates the emission behavior of Ag_2_S quantum dots under 450 nm, with the peak emission at 1210 nm and a full width at half-maximum (fwhm) of approximately 166 nm. In contrast, the Ag_2_S@SAM-B sample shows a slight red-shift in emission, peaking at 1230 nm with an fwhm of about 164 nm. This shift toward longer wavelengths is supported by HRTEM data and is attributed to the quantum confinement effect. Specifically, the particle size of Ag_2_S quantum dots increased from 6.7 to 8.3 nm after SAM surface modification, leading to the emission shift from 1210 to 1230 nm. This result highlights the broadband optical properties while avoiding interference from the water absorption peak at approximately 1450 nm, thus minimizing moisture-related disruptions during sensing operations. Overall, the surface-modified Ag_2_S quantum dots show enhanced hydrophilicity and SWIR emission, improving their compatibility with PALF for smart textile integration.

### Fluorescent Smart Textile

The successful bonding of hydrophilic Ag_2_S@SAM-B on PALF-lyocell textile fibers is essential for producing the target fluorescent smart textiles. Although physical printing methods are often convenient and cost-effective, they rely on weaker interactions between the material, which may render the luminescent particles susceptible to physicomechanical stress, making them detach easily, thus reducing their functionality. To address this issue, we employ a chemical cross-linking method using citric acid as a cross-linking agent to improve the interaction of fluorescent materials with textile fibers. This method enhances the stability and durability of its covered fluorescent properties. Figure S8a shows the FTIR spectra of PALF-lyocell yarns (pristine yarns) and the Ag_2_S@SAM-B embedded PALF-lyocell yarns (composite yarns). The emission spectrum of the composite yarns shows a strong signal at 1200 nm in the SWIR region, while the pristine yarns do not emit in this range under 450 nm excitation, further confirming the successful integration of Ag_2_S@SAM-B in the textile yarns (Figure S8b). Furthermore, SEM images comparing pristine and composite yarns (Figure S8c,d) reveal that the pristine yarns have smooth surfaces, whereas the composite yarns show particles with particle sizes around 3–5 μm on their surface.

The key functionality of fluorescent textiles lies in their ability to perform interactive functions through luminescence. To assess the effectiveness of Ag_2_S@SAM-B in such applications, we employed a cooled SWIR camera for imaging using a UV flashlight as the excitation light source. The sample was positioned approximately 30 cm from the SWIR camera, and a 1000 nm long-pass filter was applied to prevent interference from ambient light, as illustrated by the SWIR camera setup in Figure 3a. Figure 3b displays a visible light image of the pristine yarns, which are pale yellow. As expected, the pristine yarns do not appear in the SWIR images, as shown in Figure 3c. In contrast, when Ag_2_S@SAM-B is introduced into the composite yarns, they maintain a pale-yellow color under visible light, suggesting their ability to conceal markings effectively in visible light. However, under SWIR imaging, the Ag_2_S@SAM-B material becomes distinguishable, emitting a strong luminescence when irradiated with a UV flashlight. Additionally, washing fastness is a crucial factor for optical textiles. The washing procedure followed a modified American Association of Textile Chemists and Colorists (AATCC) standard procedure. After washing, the samples were air-dried. As shown in Figure S9, the infrared images before and after washing exhibit no significant difference. Based on previous experience, samples prepared using the spraying method tend to lose their optical properties after washing. However, composite yarns fabricated through chemical cross-linking retain their optical performance even after washing. These findings confirm that the composite yarns, embedded with Ag_2_S@SAM-B, function effectively as smart textile material. The textile yarns exhibit robust optical interactions in the SWIR spectrum, reinforcing their potential for advanced smart textile applications. The detailed SWIR imaging results are shown in Figure 3d,e. However, it is important to note that when optical fibers are reprocessed, unavoidable optical variations may arise due to differences in thickness, placement angle, and winding method. Therefore, AI algorithms can effectively enhance the optical interaction capabilities.

**Figure 3:**
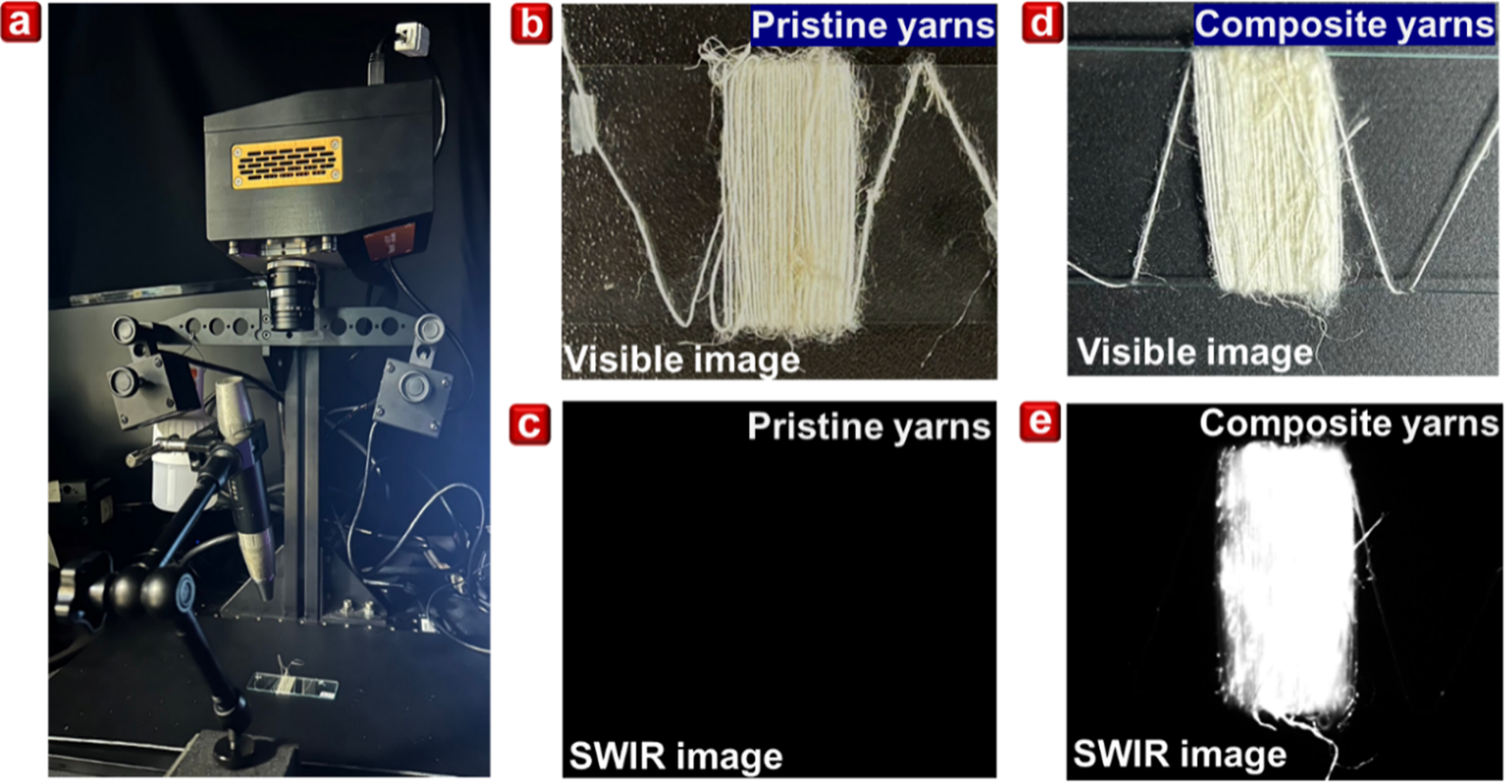
(a) Setup of the SWIR camera experiment. (b) The visible image and (c) SWIR image of pristine yarns. (d) The visible image and (e) SWIR image of composite yarns.

### SWIR Image and AI Recognition

The rapid advancement of AI has revolutionized the methods used for data collection and processing. SWIR imaging, in particular, can uncover hidden information that is not visible to the naked eye, offering powerful applications in anticounterfeiting detection and interactive features.^34,35^ The linear-scan-type hyperspectral equipment was utilized in this study to demonstrate the potential of SWIR fluorescent yarns for such recognition tasks. Each image captured has a horizontal spatial resolution of 640 pixels across 224 spectral bands ranging from 935 to 1720 nm. The composite yarns were positioned against a white background and secured with quartz glass to minimize shadows from their 3D structures, which could introduce inaccuracies. The distance between the sample platform and the hyperspectral equipment was kept at 15 cm to ensure optimal image quality, as shown in Figure 4a. In the experiment, pristine and composite yarns were twisted into a curly shape and placed on a moving platform for imaging. The resulting hyperspectral image, depicted in Figure 4b, reveals that both yarns exhibit a similar color pattern, as the hyperspectral image captures comprehensive absorption and reflection data across the wavelength range of 935–1720 nm. Specific regions from both fibers were selected to further analyze the reflection characteristics, and their reflection spectra were examined. The relative reflectance data, shown in Figure 4c, highlight a significant absorption peak at 1475 nm for both fibers, attributed to water molecules, which causes a notable decrease in relative reflectance at this wavelength. Using hyperspectral data, images were segmented across several specific wavelengths, including 952.89, 1053.43, 1150.94, 1252.41, 1350.82, 1453.22, 1552.53, and 1652.29 nm, as depicted in Figure 4d. It was observed that the images captured at 1453.22 and 1552.53 nm appeared with a darker due to water absorption. However, distinguishing pristine and composite fibers solely on the basis of these data proved challenging. To enhance the accuracy of fiber recognition, it is crucial to develop a database and incorporate AI algorithms to process the spectral data and improve the detection precision.

**Figure 4:**
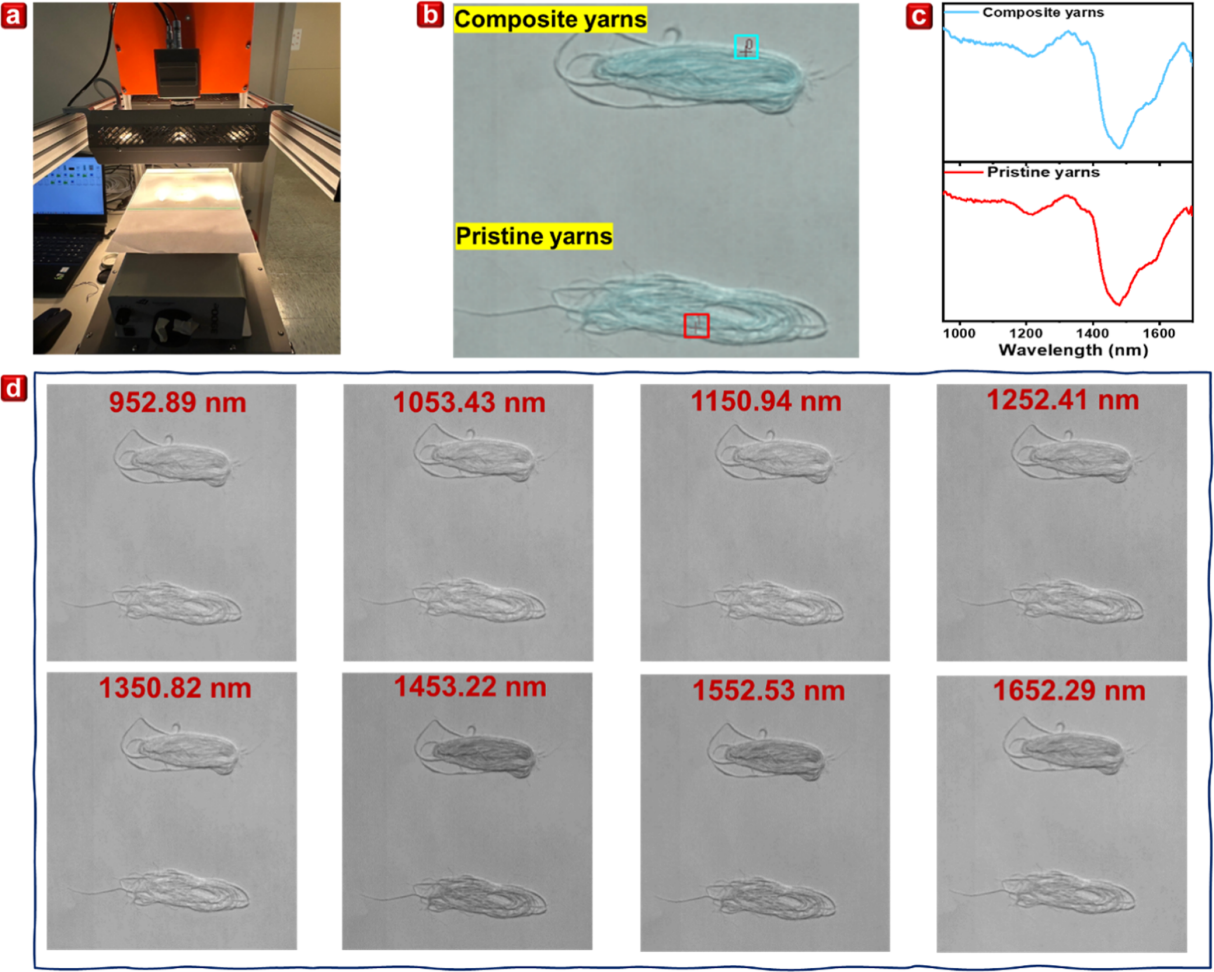
(a) Linear-scan-type hyperspectral equipment. (b) Hyperspectral image of pristine yarns and composite yarns. (c) Selected regions relatively reflective spectra from hyperspectral image. (d) Hyperspectral segment images under different wavelengths.

Integrating AI recognition technology with hyperspectral imaging commonly employs machine learning and deep learning algorithms to leverage the vast data in hyperspectral images.^36^ These algorithms are designed for pattern recognition, classification, and predictive analysis. Using machine learning techniques, models can be developed to recognize and classify objects based on features extracted from hyperspectral data. On the other hand, deep learning algorithms, particularly convolutional neural networks (CNN) and recurrent neural networks (RNN), are also prevalent in the analysis of hyperspectral images.^37,38^ These algorithms excel at learning complex feature representations and improving recognition accuracy and robustness through extensive training on large data sets of hyperspectral images. In this study, pristine and composite yarns were randomly rolled into balls with diameters of approximately 0.6 cm. We aimed to identify them within small volumes to explore the feasibility of detecting fine yarn distinctions. Figure 5a shows a visible light image of eight randomly selected yarn samples, where visual differences among the yarns are indistinguishable. The corresponding hyperspectral image is shown in Figure 5b. A sequence of algorithmic procedures is employed on the hyperspectral image to enhance the subsequent deep-learning analysis. Initially, each spectral image was processed using the spectral angle mapper (SAM) algorithm to obtain masks for the samples and background, effectively removing the background, as shown in Figure 5c. The red- and light-blue-framed areas represent pristine and composite yarns, respectively, which were used to train the database. Subsequently, image processing techniques (labeling and region props) were employed to calculate and label the contiguous regions for each sample. Afterward, the first and last 10 noisy spectral bands were removed, and the remaining data were transformed using principal components analysis (PCA) with five components (*n*_components = 5).^39^ The resulting data for each sample were (*L*, 5), where *L* corresponds to the number of spectra for a given sample. Due to the varying sizes of contiguous regions across the samples, we designed a CNN model capable of handling spectral data of different lengths.^40^ The model structure is described as follows: (1) input layer: the preprocessed sample data are input into the model. (2) Feature extraction with convolutional layers: three Conv1D layers with a kernel size of (3 × 1) and 128 filters each, followed by BatchNormalization (BN) layers, and activated using the Gaussian error linear unit (GELU) function. (3) Pooling and regularization: a MaxPool layer to retain important features and a dropout layer (ratio = 0.3) to prevent overfitting. (4) Further feature extraction: another set of three Conv1D layers with a kernel size of (3 × 1) and 256 filters each, followed by BN layers and the GELU activation function. (5) Global pooling and flattening: a GlobalMaxPool layer and a flattened layer to condense the features and prepare them for fully connected layers (6) fully connected layers: two dense layers with 256 and 128 units, respectively, using L2 regularization (ratio = 10^–5^) and the GELU activation function. Each dense layer is followed by a dropout layer (ratio = 0.3) to avoid overfitting. (7) Output layer: the final dense layer uses softmax activation to output the classification results.

**Figure 5:**
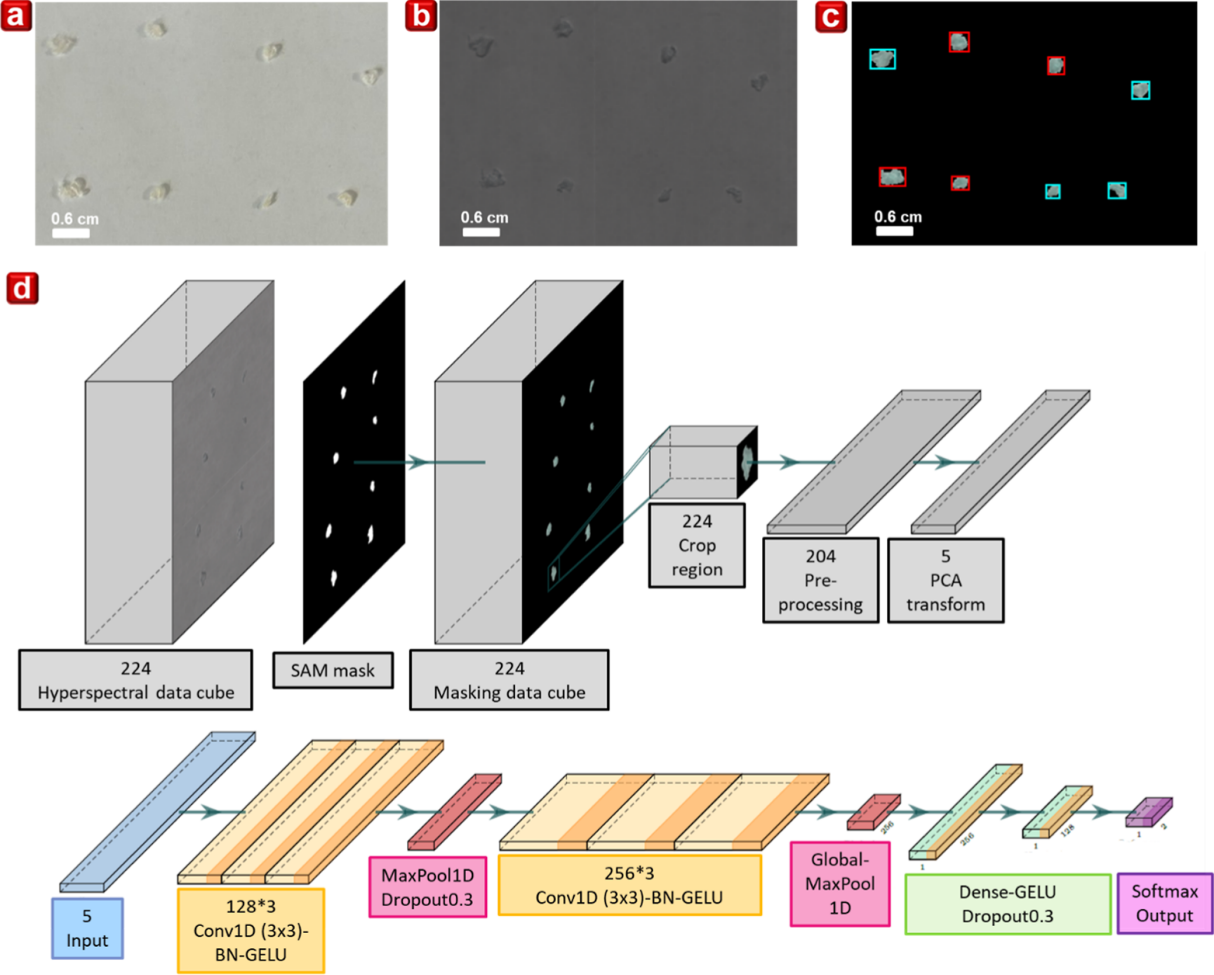
(a) Image of 0.6 cm ball-shaped fibers. (b) Hyperspectral image of ball-shaped fiber samples. (c) Modification and labeling of data. (d) The flowchart of the proposed 1D convolution neural network (CNN).

The data processing and CNN architecture used in this study are illustrated in Figure 5d and hyperparameter table also shown in Table S1.^41,42^ A total of 3 reflectance images were captured, each containing 8 samples, resulting in a total of 24 samples. The model was compiled with the Adam optimizer for training at a learning rate of 1 × 10^–4^. The loss function used was categorical_crossentropy, and the model was trained for 200 epochs. The training set consisted of 8 samples, while the test set comprised 16. The model achieved a final accuracy of 82% for detecting pristine and composite fibers. In summary, our model has the potential to predict and detect samples containing composite yarns. Even with yarn balls as small as 0.6 cm, it still demonstrates exceptionally high accuracy. This indicates that the SWIR fluorescent yarns, combining Ag_2_S@SAM-B and PALF-lyocell textile fiber, exhibit distinguishability and interactivity in optical imaging. In addition, to verify the effect of washing on the recognition efficiency, we conducted hyperspectral measurements on samples subjected to two washing cycles following the AATCC standard laundering procedure. These samples were then analyzed by using the established CNN model. The results showed that among 21 mixed measurements containing both pristine yarns and twice-washed composite yarns, 17 were correctly classified and 4 were misclassified, yielding an overall accuracy of 81%. This accuracy is very similar to that obtained with unwashed samples, indicating that the proposed composite material strategy and recognition model can maintain stable predictive performance even after at least two washing cycles (Figure S10). Although the current data volume cannot be considered big data, establishing a database with thousands of data points could significantly increase recognition accuracy, making smart textiles functional with image interaction, communication, and anticounterfeiting capabilities.

## Conclusions

In summary, we developed a scalable process for enhancing the hydrophilicity of Ag_2_S quantum dots and integrating them into PALF-lyocell yarns. The Ag_2_S QDs were stabilized by using a binary SAM shell. Ball milling yielded Ag_2_S@SAM-B particles with reduced size and improved dispersibility. HRTEM confirmed an average QD size of 8.3 ± 1.0 nm. The SAM coating increased the zeta potential from −4.45 to −8.59 mV, indicating stronger hydrophilicity. The Ag_2_S@SAM-B yarns exhibited luminescence in the SWIR region. Through chemical cross-linking, the nanocomposites were anchored to natural fibers and processed into fluorescent yarns. SWIR imaging confirmed successful signal detection from 0.6 cm yarn segments. Hyperspectral data were processed by using a 1D-CNN model that achieved 82% recognition accuracy. This approach offers a sustainable route for functionalizing textiles with optical markers. The strategy may extend to diverse fields, including image-guided sensing, anticounterfeiting, and smart packaging. It also supports future integration into AI-driven platforms for real-time information encoding and material–system interaction.

## Methods

### Materials

Diethyldithiocarbamic acid silver salt (AgS_2_CN­(CH_2_CH_3_)_2_; 99.99%), 1-dodecanethiol (C_12_H_26_S; 98%), and disec-butoxyaluminoxytriethoxysilane (C_14_H_33_AlO_6_Si; 98%) were purchased from Thermo. Carboxymethyl cellulose sodium salt (C_8_H_15_NaO_8_) and citric acid anhydrous (C_2_H_6_O; 99.5%) were purchased from Sigma. Ethanol (95% and 99%) was purchased from Echo Chemical. Toluene (ACS Reagent ≥99.5%) was purchased from UniRegion.

### Synthesis of Ag_2_S Quantum Dots

0.1 mmol of diethyldithiocarbamic acid silver salt (Ag­(DDTC)) as the precursor was weighed and 10 g of 1-dodecanethiol (DT) as the solvent was used. Both were placed in a 50 mL three-neck round-bottom flask and set up in a heating mantle with magnetic stirring. Using a Schlenk line system, an initial gas exchange at room temperature was performed to establish an inert atmosphere. The inclined double-hole stopper was connected to the vacuum line and the system evacuated for 1 min with a vacuum pump. Then, the stopper was slowly rotated to the nitrogen line for 1 min to refill with nitrogen. Each evacuation and refilling cycle constitutes one cycle, and three cycles were required. After completing the gas exchange, the stopper was turned back to the vacuum line and, under vacuum, heated to 100 °C, maintaining this temperature for 30 min to remove any moisture. Then, the stopper was turned toward the nitrogen valve, the temperature was increased to 230 °C, and the stopper was maintained for 60 min to introduce nitrogen as an inert gas. The heating mantle was removed and the three-neck round-bottom flask was allowed to cool in air until it reached room temperature. To precipitate the Ag_2_S quantum dots, 99% ethanol as an antisolvent was added to the reaction mixture at a volume ratio of 1:4 (mother liquor). The mixture was centrifuged at 10,000 rpm for 3 min. The resulting gray precipitate was Ag_2_S quantum dots, which can be dispersed in 95% ethanol for future use.

### Synthesis of Ag_2_S@SAM and the Ball-Milling Process

A dispersion of Ag_2_S quantum dots was prepared in 95% ethanol at a concentration of 10 mg/mL and placed in a 20 mL sample vial. 1.42 mmol of disec-butoxyaluminoxytriethoxysilane (DBATES) was added to the mixture. The sample vial was sealed with a serum stopper and heated in a 60 °C water bath with vigorous stirring, allowing it to react overnight. After cooling, the solution in the sample vial was mixed with toluene at a volume ratio of 1:1. It was centrifuged at 3000 rpm for 3 min to separate the supernatant, resulting in a paste-like precipitate of Ag_2_S@SAM. The sample was transferred to a sealed benchtop sulfuric acid desiccator and connected to a vacuum pump, drying it under reduced pressure. The dried particles were collected and placed in a zirconia jar containing ten zirconia balls with a diameter of 0.1 cm. A multisample rapid ball mill with a vibration frequency of 20 Hz was used to mill for 5 min to homogenize the sample. Finally, a powder sieve was used to separate and collect the powder sample, storing it in a sample vial for future use.

### Synthesis of Ag_2_S@SAM in Pineapple-Lyocell Fibers

Carboxymethyl cellulose sodium salt (CMC) was used at a concentration of 1% w/v as the dispersant and stabilizing solvent, and added to the 0.2% Ag_2_S@SAM already dispersed in 100 mL of deionized water (DI water). The mixture was sonicated for 2 h to ensure uniform mixing. The pineapple fibers were soaked in the resulting mixed solution. Then, citric acid anhydrous was added at a concentration of 10% w/w as a cross-linking agent and the solution let stand. The textile fibers were removed and heated for 6 h to completely dry. The treated pineapple fibers were blended with lyocell fibers into a carding machine until a homogeneous blend was observed. After the carding process, the blended fibers were subjected to a conventional yarn spinning line, which includes drawing, Roving, and spinning processes, resulting in Ag_2_S@SAM in pineapple-lyocell yarns.

## Supplementary Material



## Data Availability

All code and data supporting this study, including raw and processed spectra, trained weights, inference scripts, and environment files, are publicly available at Zenodo (DOI: 10.5281/zenodo.17239584) and GitHub (https://github.com/MHlab-user/Optical-Labeling-with-Artificial-Intelligence-Using-Infrared-Responsive-Functional-Textiles).

## Notes

Characterization, method, crystal structure of Ag_2_S, SEM, and particle size distribution of Ag_2_S, EDS mapping images, lattice fringes of the Ag_2_S in HRTEM, detailed information on FTIR spectra, absorption spectra of Ag_2_S, and the image before and after washing of composite yarns (PDF)
