# Supplementary material for: Optical Labeling with Artificial Intelligence Using Infrared-Responsive Functional Textiles
Source: ACS Appl Mater Interfaces. 2025 Oct 11;17(42):58532–9. doi: 10.1021/acsami.5c13595 (PMC12557207; doi:10.1021/acsami.5c13595)
Supplement: Supplementary file 1 [file am5c13595_si_001.pdf]

Supporting Information

Optical Labeling with Artificial Intelligence Using Infrared-Responsive Functional Textiles

Yi-Ting Tsai[†,#], Chi-Wei Wu[†,§,#], Ren-Jei Chung[§,#], Gabriel Nicolo A. De Guzman[Φ], Guan-Jie Li[‡], Julius L. Leaño Jr.[Φ], and Mu-Huai Fang[†,]*

[#]These authors contributed equally to this work.

†Research Center for Applied Sciences, Academia Sinica, Taipei 11529, Taiwan.

§Department of Chemical Engineering and Biotechnology, National Taipei University of Technology, Taipei 10608, Taiwan

Φ Philippine Textile Research Institute, Department of Science and Technology, Taguig City 1631, Philippines

‡ Isuzu Optics Cooperation, Hsinchu 30288, Taiwan

Corresponding Author

*Mu-Huai Fang – E-mail: fangmuhuai@gate.sinica.edu.tw

CHARACTERIZATION:

High-resolution synchrotron XRD patterns were measured at the National Synchrotron Radiation Research Center (NSRRC, Taiwan) TLS 01C2 beamline. Under the conditions of 16 keV voltage and a wavelength of 0.77491 Å, the scanning range of the crystal plane and the 2θ range is between 5° and 45°. The high-resolution TEM images with EDS mapping are measured by JEM-2100F (JEOL) and analyzed the particle distribution by ImageJ software. The scanning electron microscope (SEM) images are measured by ThermoFisher Phenom Pharos. Fourier transform infrared spectroscopy (FTIR) is characterized by Bruker VERTEX 70v with range between 500–4000 cm^{-1}. Manual hydraulic press (Panchum) is used for pressing to 10 mm pellet. Zeta potential analysis is measured by Malvern Panalytical Zetasizer ZS90. Photoluminescence (PL) spectra of Ag_2S were measured with a Horiba Fluorolog-QM with 1000 nm color filter. Spectrofluorometer equipped with a 75 W xenon lamp as an excitation source and an R5509 Hamamatsu photomultiplier as a detector. UV-visible/NIR absorption spectrophotometers (Jasco V-670 ABS) equipped with deuterium lamp (for 190–350 nm) and halogen lamp (for 350–2700 nm). The SWIR image captured by Ninox 640II cooled camera.-The SWIR hyperspectral setup includes the Specim FX17e, three halogen lamps, and the LabScanner 40 cm x 20 cm moving platform,

measuring with 3 ms integral time. (supplied by Isuzu Optics).

All analyses were conducted using Python 3.12.0 (64-bit) together with the following packages and versions:

**Package Name**	**Version**	**Description**
numpy	1.26.4	Numerical computing
matplotlib	3.10.5	Data visualization and plotting
scikit-learn	1.7.1	Machine learning, including PCA
scikit-image	0.25.2	Image processing, region segmentation
spectral	0.24	Hyperspectral data analysis and I/O
tensorflow	2.20.0	Deep learning framework
keras	3.11.3	Neural network high-level API for TensorFlow
pillow	11.3.0	Image file input/output and processing

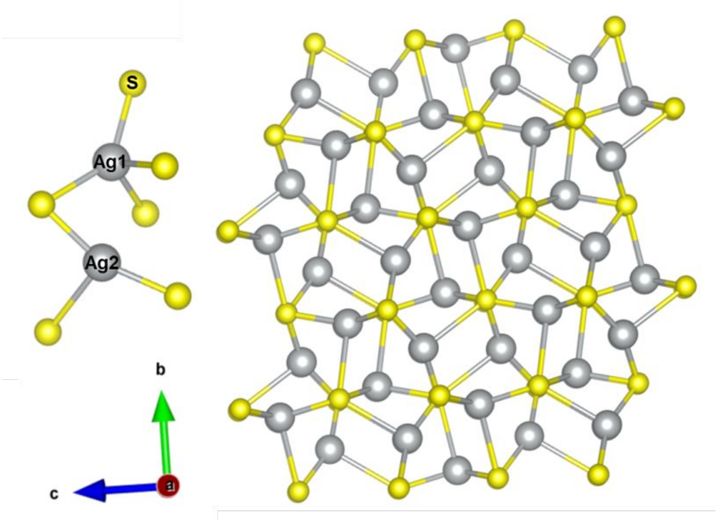

Figure S1. The crystal structure of Ag_2S. The first Ag(1) site occupied a 4-coordinate tetrahedron with four equivalent S^{2-} ligands with Ag–S bond lengths ranging from 2.50 to 2.99 Å. The second Ag(2) site occupied a 3-coordinate trigonal plane with three equivalent S^{2-} ligands, resulting in Ag–S bond distances ranging from 2.54 to 3.06 Å.

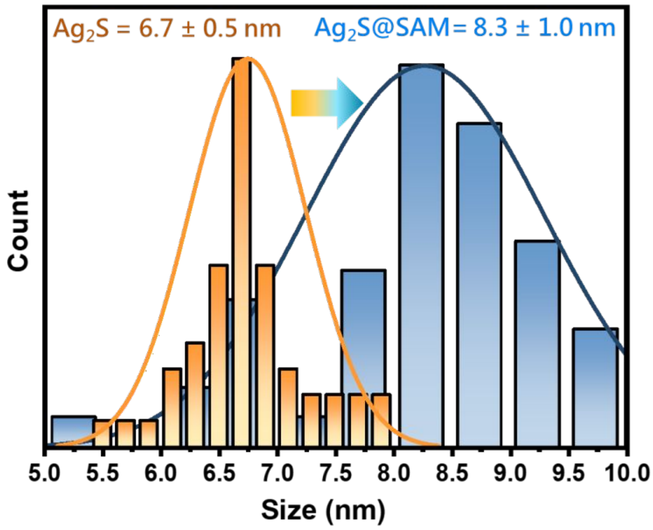

Figure S2. The particle size distribution of Ag_2S quantum dots and Ag_2S@SAM.

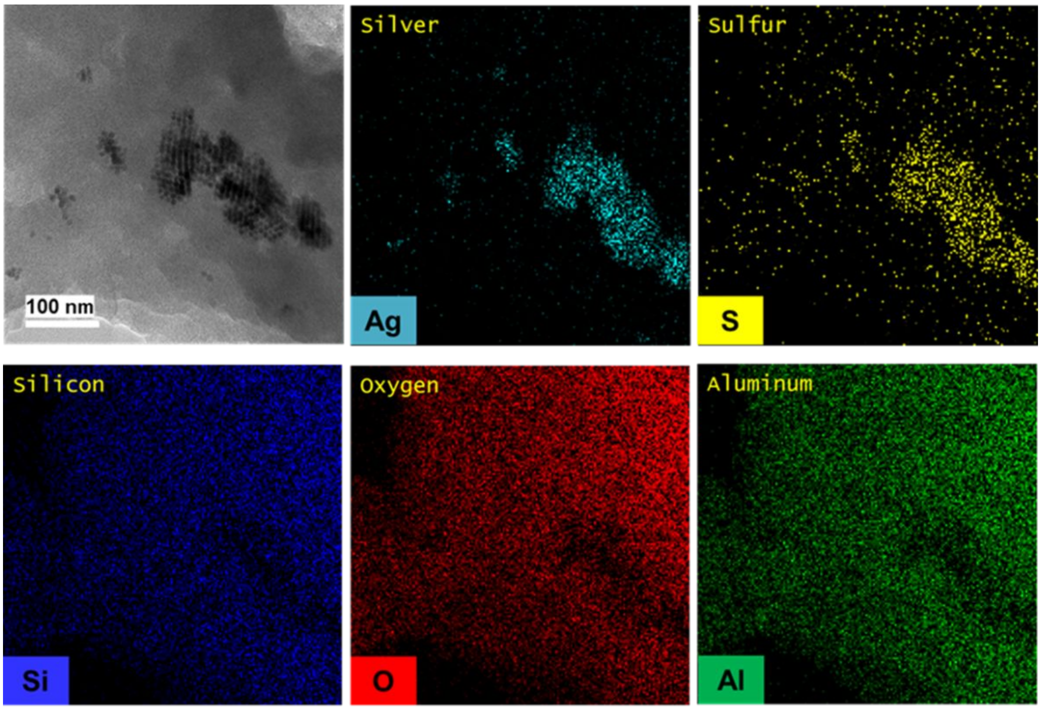

Figure S3. EDS mapping images of silver, sulfur, silicon, oxygen, and aluminum.

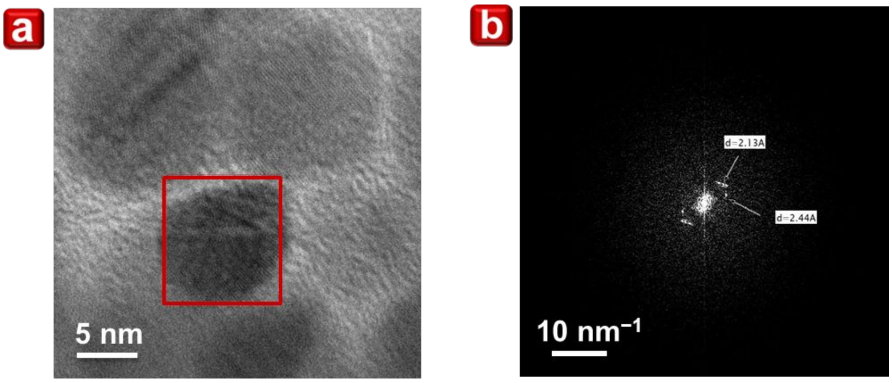

Figure S4. (a) Lattice fringes of the Ag_2S in HRTEM. (b) Fast Fourier-transform image from red frame area in a. The lattice spacings (*d*-spacings) of 2.13 Å and 2.44 Å, corresponding to the (122) and (121) crystal planes of the Ag_2S, respectively.

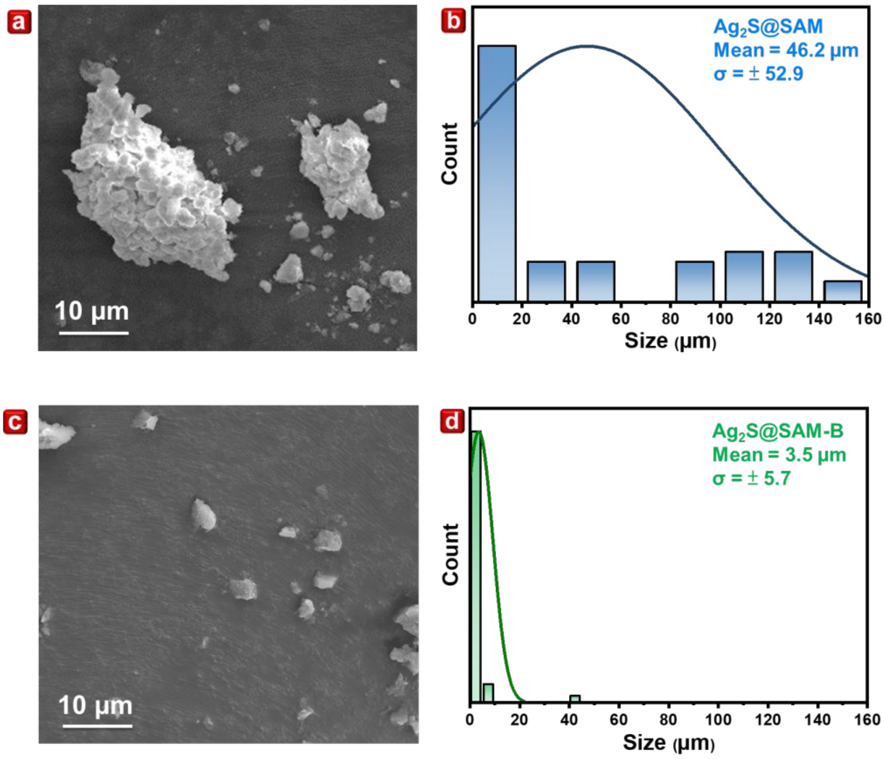

Figure S5. Scanning electron microscopy images of (a) Ag_2S@SAM and (c) Ag_2S@SAM-B. Particle size analysis of (b) Ag_2S@SAM and (d) after ball-milling Ag_2S@SAM-B.

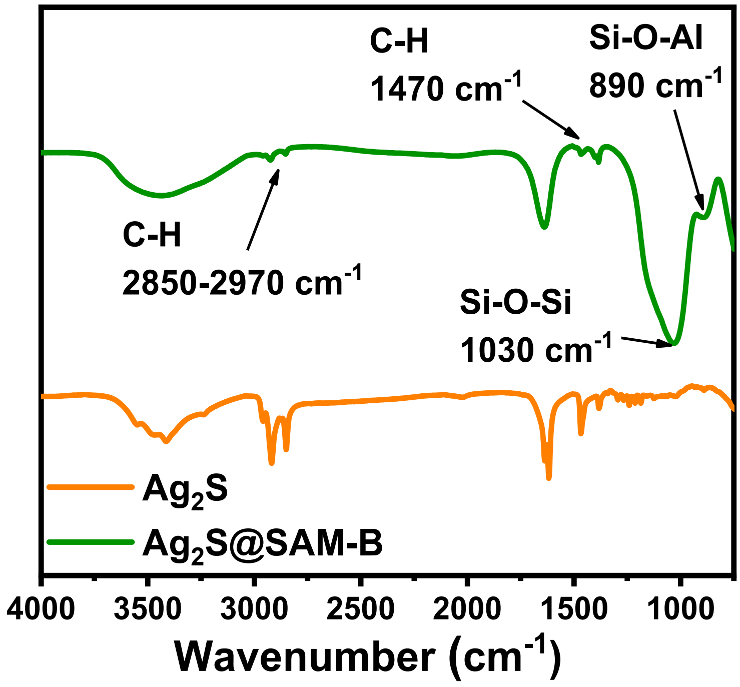

Figure S6. FTIR spectra of Ag_2S and Ag_2S@SAM-B. Compared to the FTIR spectrum of pure Ag_2S QDs (green line), the C–H stretching signals at 2850–2970 cm^{-1} and C–H bending signals at 1470 cm^{-1} significantly weaken after being coated with SAM. In addition, a new characteristic peak corresponding to the Si–O–Si functional group appeared at 1030 cm^{-1}, while the Si–O–Al functional group was detected at 890 cm^{-1}, as shown by the orange line.

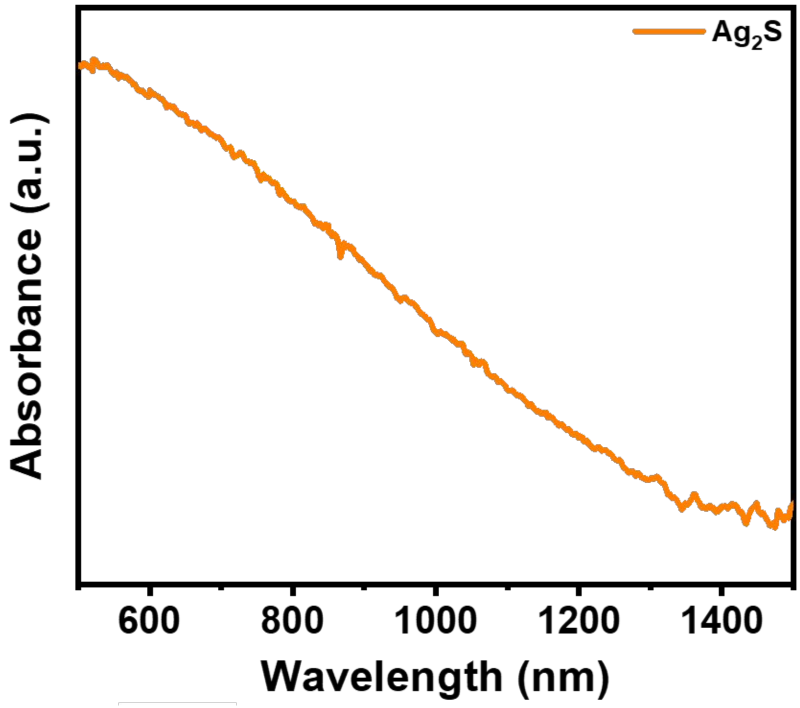

Figure S7. Absorption spectra of Ag_2S in the spectral range of 500–1500 nm.

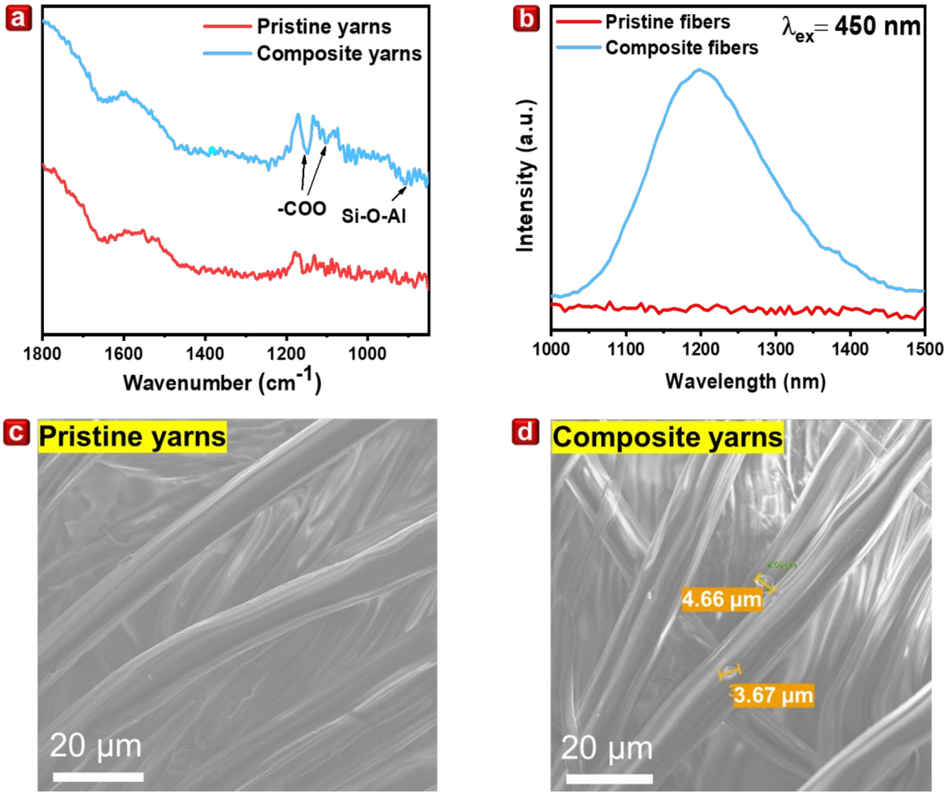

Figure S8. (a) The composite yarns show ester bond signals at 1248 and 1102 cm^{-1} (−COO stretching), formed via esterification between the −COOH of citric acid and the −OH of CMC. A peak at 893 cm^{-1} confirms the presence of the Si−O−Al group from Ag_2S@SAM-B. (b) PL spectra of pristine yarns and composite yarns under 450 nm excitation. SEM image of (c) pristine and (d) composite yarns. These particle sizes align with the size distribution of Ag_2S@SAM-B materials, as shown in Figure S5c, suggesting the successful embedding of Ag_2S@SAM-B onto the textile yarns.

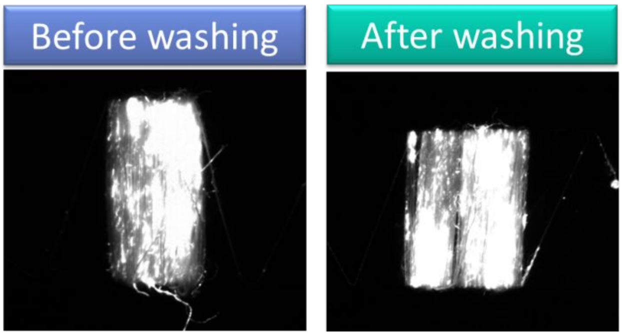

Figure S9. Infrared image before and after washing composite yarns. Ag_2S@SAM-B infused yarns were stapled in a 4" x 2" cotton fabric and were placed in a canister alongside 10 steel balls and 200 mL of 0.37% Triton-X solution. The canister was then subjected to a washing simulation using a B-5 AATCC Launder-Ometer for 1 hr at 50 ^{o}C.

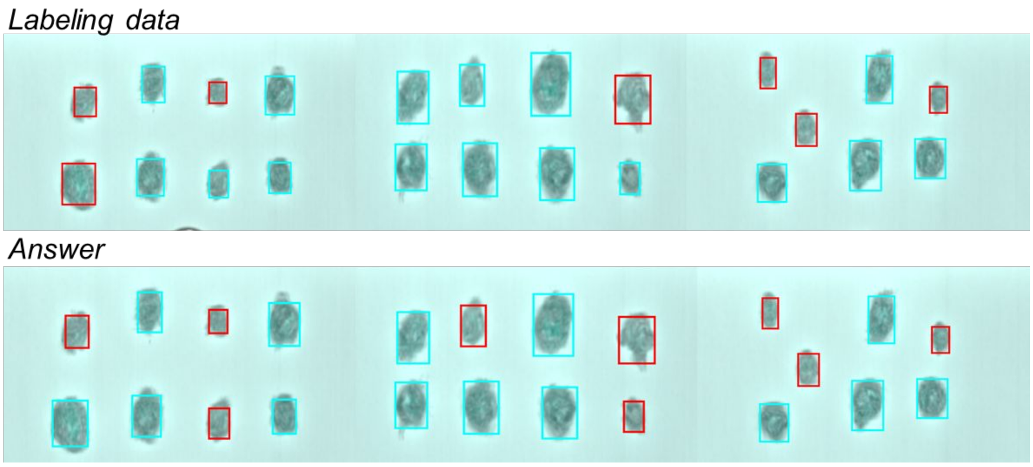

Figure S10. Classification results of pristine yarns and twice-washed composite yarns using the original CNN model. The top panels show the predicted labels for 21 test samples, where red boxes correspond to pristine yarns and blue boxes to twice-washed composite yarns. The bottom panels present the ground-truth labels. A comparison indicates that four samples were misclassified, yielding an

overall recognition accuracy of 81%.

Table S1. Neural Network Architecture

Layer Type	Number of Filters / Units	Kernel Size	Activation Function	Other Details
InputLayer	-	-	-	Input shape (None, feature_dim)
Conv1D (Block 1)	128	3	GELU	BatchNormalization + Activation repeated 3 times
MaxPool1D	-	-	-	Pooling after first Conv1D block
Dropout	-	-	-	Dropout ratio 0.3
Conv1D (Block 2)	256	3	GELU	BatchNormalization + Activation repeated 3 times
GlobalMaxPool1D	-	-	-	Global pooling before dense layers
Flatten	-	-	-	Flatten output to 1D vector
Dense Layer 1	256	-	GELU	L2 regularization applied
Dropout	-	-	-	Dropout ratio 0.3
Dense Layer 2	128	-	GELU	L2 regularization applied
Dropout	-	-	-	Dropout ratio 0.3
Output Layer (Dense)	2	-	Softmax	Two-class classification
